# Supplementary material for: Comparison and Harmonization of Different Semi-Automated and Automated qRT-PCR Assays in the Assessment of SARS-CoV-2
Source: Viruses. 2022 Oct 12;14(10):2239. doi: 10.3390/v14102239 (PMC9610219; doi:10.3390/v14102239)
Supplement: Supplementary file 1 [file viruses-14-02239-s001.zip › viruses-1914705-Suppl. Material.pdf]

Suppl. Material

Supplementary Table S1: Based on the results from our reference method on the Alinity m, sensitivity, positive predictive value (PPV), specificity and negative predictive value (NPV) were calculated for cobas® 6800, GeneXpert, genesig® and RIDA®GENE.

Assay	n	Overall Positives	Correct Positives	Sensitivity / PPV (%)	Overall Negatives	Correct Negatives	Specificity / NPV (%)
**cobas® 6800**	**85**	50	50	98.04% / 100.0%	35	34	100.0% / 97.14%
**GeneXpert**	**85**	51	51	100.0% / 100.0%	34	34	100.0% / 100.0%
**genesig®**	**85**	36	36	70.59% / 100.0%	49	34	100.0% / 69.39%
**RIDA®GENE**	**85**	46	46	90.20% / 100.0%	39	34	100.0% / 87.18%

Supplementary Table S2: Results of measurements on Alinity m, cobas® 6800, GeneXpert, genesig® and RIDA®GENE. Ct values and corresponding viral loads as calculated by standard curves are displayed. n=85. neg=negative.

	Alinity m				cobas® 6800				
**Sample No.**	**Ct RdRp- & N-Gene**	**Isolation Ct-value**	**Viral Load [copies/ml]**	**Isolation Viral Load**	**Ct ORF1a/b**	**Isolation Ct-value**	**Ct E-Gene**	**Viral Load [copies/ml]**	**Isolation Viral Load**
**1**	33.38	no	6.20E+02	no	32.84	no	34.46	3.43E+03	no
**2**	20.36	yes	2.32E+06	yes	24.81	yes	25.44	3.83E+06	yes
**3**	32.04	no	1.45E+03	no	33.4	no	34.36	2.10E+03	no
**4**	29.34	yes	7.96E+03	no	31.6	no	32.88	1.01E+04	no
**5**	35.01	no	2.21E+02	no	34.61	no	36.45	7.29E+02	no
**6**	16.31	yes	3.00E+07	yes	19.94	yes	20.71	2.70E+08	yes
**7**	19.83	yes	3.25E+06	yes	23.63	yes	24.06	1.07E+07	yes
**8**	19.97	yes	2.97E+06	yes	23.23	yes	23.66	1.52E+07	yes
**9**	19.83	yes	3.25E+06	yes	23.68	yes	24.37	1.03E+07	yes
**10**	23.7	yes	2.81E+05	no	27.59	yes	28.2	3.37E+05	no
**11**	20.76	yes	1.80E+06	yes	24.07	yes	24.72	7.31E+06	yes
**12**	22.3	yes	6.82E+05	no	25.04	yes	25.71	3.13E+06	yes
**13**	27.98	yes	1.88E+04	no	30.75	no	31.64	2.13E+04	no
**14**	14.93	yes	7.18E+07	yes	18.77	yes	19.34	7.51E+08	yes
**15**	25.4	yes	9.61E+04	no	28.27	yes	28.7	1.86E+05	no
**16**	17.68	yes	1.26E+07	yes	21.04	yes	21.81	1.03E+08	yes
**17**	17.09	yes	1.83E+07	yes	21.04	yes	21.6	1.03E+08	yes
**18**	17.03	yes	1.91E+07	yes	20.39	yes	20.95	1.82E+08	yes
**19**	25.94	yes	6.83E+04	no	29.47	yes	30.32	6.52E+04	no

**20**	13.04	yes	2.37E+08	yes	16.34	yes	17.12	6.28E+09	yes
**21**	24.59	yes	1.60E+05	no	28.24	yes	29.01	1.91E+05	no
**22**	33.56	no	5.53E+02	no	32.42	no	34.64	4.95E+03	no
**23**	19.86	yes	3.19E+06	yes	23.98	yes	24.88	7.90E+06	yes
**24**	29.73	yes	6.22E+03	no	31.88	no	32.84	7.93E+03	no
**25**	22.62	yes	5.57E+05	no	26.2	yes	26.88	1.14E+06	yes
**26**	neg	no		no	neg	no	neg		no
**27**	30.22	no	4.57E+03	no	31.66	no	33.22	9.61E+03	no
**28**	25.64	yes	8.26E+04	no	29.14	yes	29.7	8.69E+04	no
**29**	16.95	yes	2.00E+07	yes	21.94	yes	22.35	4.70E+07	yes
**30**	13.27	yes	2.05E+08	yes	19.39	yes	19.33	4.37E+08	yes
**31**	25.42	yes	9.49E+04	no	28.83	yes	29.73	1.14E+05	no
**32**	19.52	yes	3.95E+06	yes	24.58	yes	25.53	4.68E+06	yes
**33**	26.36	yes	5.24E+04	no	29.99	yes	31.02	4.14E+04	no
**34**	22.76	yes	5.10E+05	no	26.29	yes	27.21	1.05E+06	yes
**35**	20.21	yes	2.55E+06	yes	23.6	yes	23.92	1.10E+07	yes
**36**	31.74	no	1.75E+03	no	32.44	no	33.62	4.86E+03	no
**37**	35.05	no	2.16E+02	no	neg	no	35.39		no
**38**	neg	no		no	neg	no	neg		no
**39**	30.64	no	3.50E+03	no	31.56	no	33	1.05E+04	no
**40**	neg	no		no	neg	no	neg		no
**41**	17.48	yes	1.43E+07	yes	21.21	yes	21.63	8.90E+07	yes
**42**	22.12	yes	7.64E+05	no	25.42	yes	26.12	2.25E+06	yes
**43**	22.05	yes	7.98E+05	no	24.83	yes	25.45	3.76E+06	yes
**44**	neg	no		no	neg	no	neg		no
**45**	17.72	yes	1.23E+07	yes	21.42	yes	22.08	7.41E+07	yes

**46**	30.77	no	3.23E+03	no	31.13	no	32.65	1.53E+04	no
**47**	35.43	no	1.70E+02	no	34.62	no	36.5	7.23E+02	no
**48**	18.23	yes	8.92E+06	yes	21.66	yes	22.23	6.00E+07	yes
**49**	21.53	yes	1.11E+06	yes	25.3	yes	25.86	2.49E+06	yes
**50**	19.61	yes	3.73E+06	yes	22.38	yes	23.17	3.20E+07	yes
**51**	32.22	no	1.29E+03	no	32.38	no	34	5.12E+03	no
**52**	25.48	yes	9.13E+04	no	28.89	yes	29.85	1.08E+05	no
**53**	39.17	no	1.60E+01	no	neg	no	neg		no
**54**	34.67	no	2.74E+02	no	neg	no	34.59		no
**55**	31.31	no	2.29E+03	no	32.42	no	35.05	4.95E+03	no
**56**	neg	no		no	neg	no	neg		no
**57**	neg	no		no	neg	no	neg		no
**58**	neg	no		no	neg	no	neg		no
**59**	neg	no		no	neg	no	neg		no
**60**	neg	no		no	neg	no	neg		no
**61**	neg	no		no	neg	no	neg		no
**62**	neg	no		no	neg	no	neg		no
**63**	neg	no		no	neg	no	neg		no
**64**	neg	no		no	neg	no	neg		no
**65**	neg	no		no	neg	no	neg		no
**66**	neg	no		no	neg	no	neg		no
**67**	neg	no		no	neg	no	neg		no
**68**	neg	no		no	neg	no	neg		no
**69**	neg	no		no	neg	no	neg		no
**70**	neg	no		no	neg	no	neg		no
**71**	neg	no		no	neg	no	neg		no

**72**	neg	no		no	neg	no	neg		no
**73**	neg	no		no	neg	no	neg		no
**74**	neg	no		no	neg	no	neg		no
**75**	neg	no		no	neg	no	neg		no
**76**	neg	no		no	neg	no	neg		no
**77**	neg	no		no	neg	no	neg		no
**78**	neg	no		no	neg	no	neg		no
**79**	neg	no		no	neg	no	neg		no
**80**	neg	no		no	neg	no	neg		no
**81**	neg	no		no	neg	no	neg		no
**82**	neg	no		no	neg	no	neg		no
**83**	neg	no		no	neg	no	neg		no
**84**	neg	no		no	neg	no	neg		no
**85**	neg	no		no	neg	no	neg		no

	**GeneXpert**					**genesig®**			
**Sample No.**	**Ct N2-Gene**	**Isolation Ct-value**	**Ct E-Gene**	**Viral Load [copies/ml]**	**Isolation Viral Load**	**Ct ORF1a/b**	**Isolation Ct-value**	**Viral Load [copies/ml]**	**Isolation Viral Load**
**1**	38.1	no	35.1	5.85E+02	no	neg	no		no
**2**	25.5	yes	23.4	2.82E+06	yes	30.75	no	6.16E+05	no
**3**	37.1	no	33.5	1.15E+03	no	neg	no		no
**4**	34.7	no	31.6	5.77E+03	no	36.15	no	1.39E+04	no
**5**	39.6	no	35.5	2.13E+02	no	neg	no		no
**6**	19.5	yes	18	1.60E+08	yes	27.94	yes	4.29E+06	yes
**7**	23.3	yes	21.6	1.24E+07	yes	31.51	no	3.66E+05	no
**8**	23.8	yes	21.6	8.85E+06	yes	30.94	no	5.40E+05	no

**9**	24.2	yes	22.4	6.76E+06	yes	31.48	no	3.73E+05	no
**10**	27.7	yes	26.2	6.41E+05	no	36.57	no	2.16E+04	no
**11**	24.4	yes	22.8	5.91E+06	yes	32.32	no	2.09E+06	yes
**12**	26.6	yes	24.2	1.34E+06	yes	32.08	no	2.46E+05	no
**13**	33.8	no	30.9	1.06E+04	no	37.73	no	3.87E+03	no
**14**	19.4	yes	17.4	1.71E+08	yes	25.23	yes	2.78E+07	yes
**15**	29.3	yes	27.4	2.18E+05	no	36.22	no	2.80E+04	no
**16**	21.4	yes	19.8	4.45E+07	yes	neg	no		no
**17**	20	yes	18.2	1.14E+08	yes	26.88	yes	8.91E+06	yes
**18**	20.4	yes	18.3	8.72E+07	yes	25.99	yes	1.66E+07	yes
**19**	30.9	no	28.4	7.44E+04	no	35.21	no	2.81E+04	no
**20**	16.2	yes	13.4	1.47E+09	yes	20.79	yes	5.95E+08	yes
**21**	29.5	yes	27	1.91E+05	no	34.91	no	3.48E+04	no
**22**	37.2	no	35.3	1.07E+03	no	neg	no		no
**23**	22.6	yes	21.1	1.98E+07	yes	29.56	yes	1.31E+06	yes
**24**	35.6	no	32.5	3.15E+03	no	36.81	no	8.32E+03	no
**25**	27.8	yes	26	5.99E+05	no	33.81	no	7.51E+04	no
**26**	neg	no	neg		no	neg	no		no
**27**	33.6	no	31.4	1.21E+04	no	neg	no		no
**28**	30.4	no	28.3	1.04E+05	no	36.15	no	1.39E+04	no
**29**	20.9	yes	19.5	6.23E+07	yes	28.3	yes	3.35E+06	yes
**30**	16.8	yes	16.4	9.84E+08	yes	25.81	yes	1.87E+07	yes
**31**	29.7	yes	27.3	1.67E+05	no	35.73	no	1.91E+04	no
**32**	23.7	yes	21.4	9.46E+06	yes	30.97	no	5.33E+06	yes
**33**	31.3	no	29.1	5.68E+04	no	35.88	no	1.71E+04	no
**34**	27.9	yes	25.9	5.60E+05	no	33.34	no	1.03E+05	no

**35**	24.7	yes	22.3	4.83E+06	yes	30.97	no	5.30E+06	yes
**36**	36.8	no	34.1	1.40E+03	no	neg	no		no
**37**	38.2	no	34.5	5.47E+02	no	neg	no		no
**38**	neg	no	neg		no	neg	no		no
**39**	34.1	no	31.4	8.64E+03	no	neg	no		no
**40**	neg	no	neg		no	neg	no		no
**41**	22.2	yes	20.5	2.60E+07	yes	29.63	yes	1.34E+06	yes
**42**	24	yes	22.6	7.73E+06	yes	30.7	no	6.39E+05	no
**43**	28.4	yes	23.6	4.00E+05	no	32.97	no	1.33E+05	no
**44**	neg	no	neg		no	neg	no		no
**45**	22.8	yes	20.3	1.73E+07	yes	28.84	yes	2.31E+06	yes
**46**	34.7	no	31.5	5.77E+03	no	neg	no		no
**47**	41	no	37.8	8.31E+01	no	neg	no		no
**48**	22.9	yes	20.5	1.62E+07	yes	29.55	yes	1.41E+06	yes
**49**	25.5	yes	23.6	2.82E+06	yes	32.04	no	2.53E+06	yes
**50**	23.4	yes	21.1	1.16E+07	yes	29.04	yes	2.01E+06	yes
**51**	37.3	no	33.8	1.00E+03	no	neg	no		no
**52**	30.5	no	28.6	9.74E+04	no	35.46	no	2.35E+04	no
**53**	41	no	neg	8.31E+01	no	neg	no		no
**54**	36	no	33.8	2.40E+03	no	neg	no		no
**55**	34.7	no	32.3	5.77E+03	no	neg	no		no
**56**	neg	no	neg		no	neg	no		no
**57**	neg	no	neg		no	neg	no		no
**58**	neg	no	neg		no	neg	no		no
**59**	neg	no	neg		no	neg	no		no
**60**	neg	no	neg		no	neg	no		no

**61**	neg	no	neg		no	neg	no		no
**62**	neg	no	neg		no	neg	no		no
**63**	neg	no	neg		no	neg	no		no
**64**	neg	no	neg		no	neg	no		no
**65**	neg	no	neg		no	neg	no		no
**66**	neg	no	neg		no	neg	no		no
**67**	neg	no	neg		no	neg	no		no
**68**	neg	no	neg		no	neg	no		no
**69**	neg	no	neg		no	neg	no		no
**70**	neg	no	neg		no	neg	no		no
**71**	neg	no	neg		no	neg	no		no
**72**	neg	no	neg		no	neg	no		no
**73**	neg	no	neg		no	neg	no		no
**74**	neg	no	neg		no	neg	no		no
**75**	neg	no	neg		no	neg	no		no
**76**	neg	no	neg		no	neg	no		no
**77**	neg	no	neg		no	neg	no		no
**78**	neg	no	neg		no	neg	no		no
**79**	neg	no	neg		no	neg	no		no
**80**	neg	no	neg		no	neg	no		no
**81**	neg	no	neg		no	neg	no		no
**82**	neg	no	neg		no	neg	no		no
**83**	neg	no	neg		no	neg	no		no
**84**	neg	no	neg		no	neg	no		no
**85**	neg	no	neg		no	neg	no		no

	RIDA®GENE			
**Sample No.**	**Ct E-Gene**	**Isolation Ct-value**	**Viral Load [copies/ml]**	**Isolation Viral Load**
**1**	40	no	8.61E+02	no
**2**	28.68	yes	2.20E+06	yes
**3**	40	no	8.61E+02	no
**4**	35.85	no	1.53E+03	no
**5**	neg	no		no
**6**	25	yes	2.83E+07	yes
**7**	28.49	yes	2.51E+06	yes
**8**	28.34	yes	2.79E+06	yes
**9**	28.73	yes	2.13E+06	yes
**10**	32.73	no	1.33E+05	no
**11**	28.94	yes	1.83E+06	yes
**12**	29.56	yes	1.20E+06	yes
**13**	37.01	no	6.84E+03	no
**14**	23.09	yes	1.06E+08	yes
**15**	33.2	no	9.52E+04	no
**16**	30.99	no	4.43E+05	no
**17**	24.16	yes	5.04E+07	yes
**18**	24.29	yes	4.63E+07	yes
**19**	33.87	no	6.05E+04	no
**20**	18.91	yes	1.92E+09	yes
**21**	33.14	no	9.97E+04	no
**22**	40	no	8.61E+02	no
**23**	27.08	yes	6.69E+06	yes

**24**	40	no	8.61E+02	no
**25**	31.56	no	2.98E+05	no
**26**	neg	no		no
**27**	38.01	no	3.42E+03	no
**28**	34.99	no	2.77E+04	no
**29**	25.46	yes	2.04E+07	yes
**30**	22.07	yes	2.16E+08	yes
**31**	33.92	no	5.84E+04	no
**32**	27.13	yes	6.45E+06	yes
**33**	34.1	no	5.15E+04	no
**34**	31.11	no	4.09E+05	no
**35**	28.62	yes	2.30E+06	yes
**36**	38.55	no	2.36E+03	no
**37**	neg	no		no
**38**	neg	no		no
**39**	39.46	no	1.25E+03	no
**40**	neg	no		no
**41**	26.32	yes	1.13E+07	yes
**42**	28.27	yes	2.92E+06	yes
**43**	29.79	yes	1.02E+06	yes
**44**	neg	no		no
**45**	25.9	yes	1.52E+07	yes
**46**	40	no	8.61E+02	no
**47**	neg	no		no
**48**	26.55	yes	9.56E+06	yes
**49**	29.54	yes	1.21E+06	yes

**50**	27.04	yes	6.85E+06	yes
**51**	neg	no		no
**52**	34.16	no	4.94E+04	no
**53**	neg	no		no
**54**	40	no	8.61E+02	no
**55**	36.83	no	7.72E+03	no
**56**	neg	no		no
**57**	neg	no		no
**58**	neg	no		no
**59**	neg	no		no
**60**	neg	no		no
**61**	neg	no		no
**62**	neg	no		no
**63**	neg	no		no
**64**	neg	no		no
**65**	neg	no		no
**66**	neg	no		no
**67**	neg	no		no
**68**	neg	no		no
**69**	neg	no		no
**70**	neg	no		no
**71**	neg	no		no
**72**	neg	no		no
**73**	neg	no		no
**74**	neg	no		no
**75**	neg	no		no

**76**	neg	no		no
**77**	neg	no		no
**78**	neg	no		no
**79**	neg	no		no
**80**	neg	no		no
**81**	neg	no		no
**82**	neg	no		no
**83**	neg	no		no
**84**	neg	no		no
**85**	neg	no		no

Supplementary Table S3: Results of standard curve measurements. Defined viral loads were analyzed on the respective systems. Ct values and the corresponding viral loads are shown. Viral loads below 10^3 were not detectable for genesig® and RIDA®GENE.

	Ct values				
**copies/ml**	**Alinity m**	**cobas® 6800**	**GeneXpert**	**genesig®**	**RIDA®GENE**
**1.00E+07**	17.95	23.77	23.62	27.70	26.43
**1.00E+06**	21.61	26.41	27.04	31.04	29.75
**1.00E+05**	25.99	29.86	30.46	34.38	33.07
**1.00E+04**	29.10	32.36	33.88	37.71	36.40
**1.00E+03**	32.30	34.20	37.30	n.a.	n.a.
**1.00E+02**	36.37	36.88	40.72	n.a.	n.a.

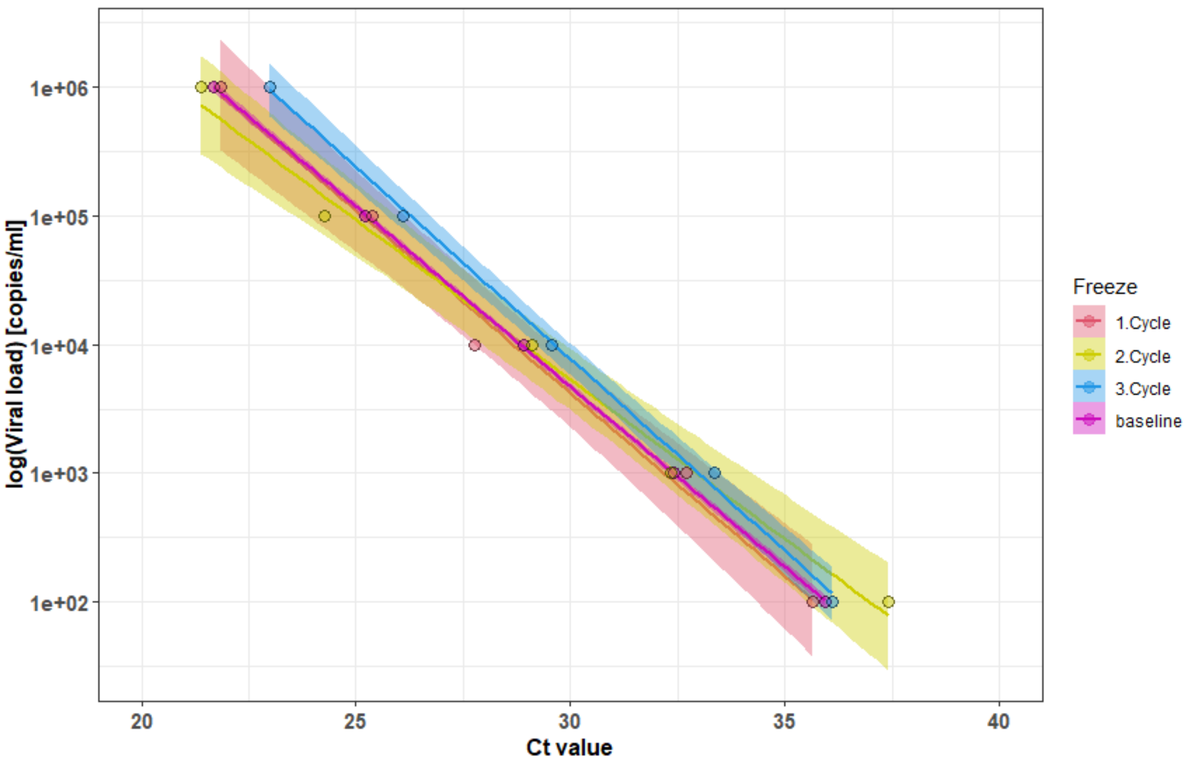

Supplementary Figure S1: Impact of freeze/thaw cycles on standard curve generation. Standard curves were generated via serial dilution measurements of a sample with a known SARS-CoV-2 concentration using our reference method on the Alinity m. This was done at baseline and following three freeze/thaw cycles (12 hours freezing at -80°C for each cycle). Standard curves are shown with the respective 95% confidence intervals.

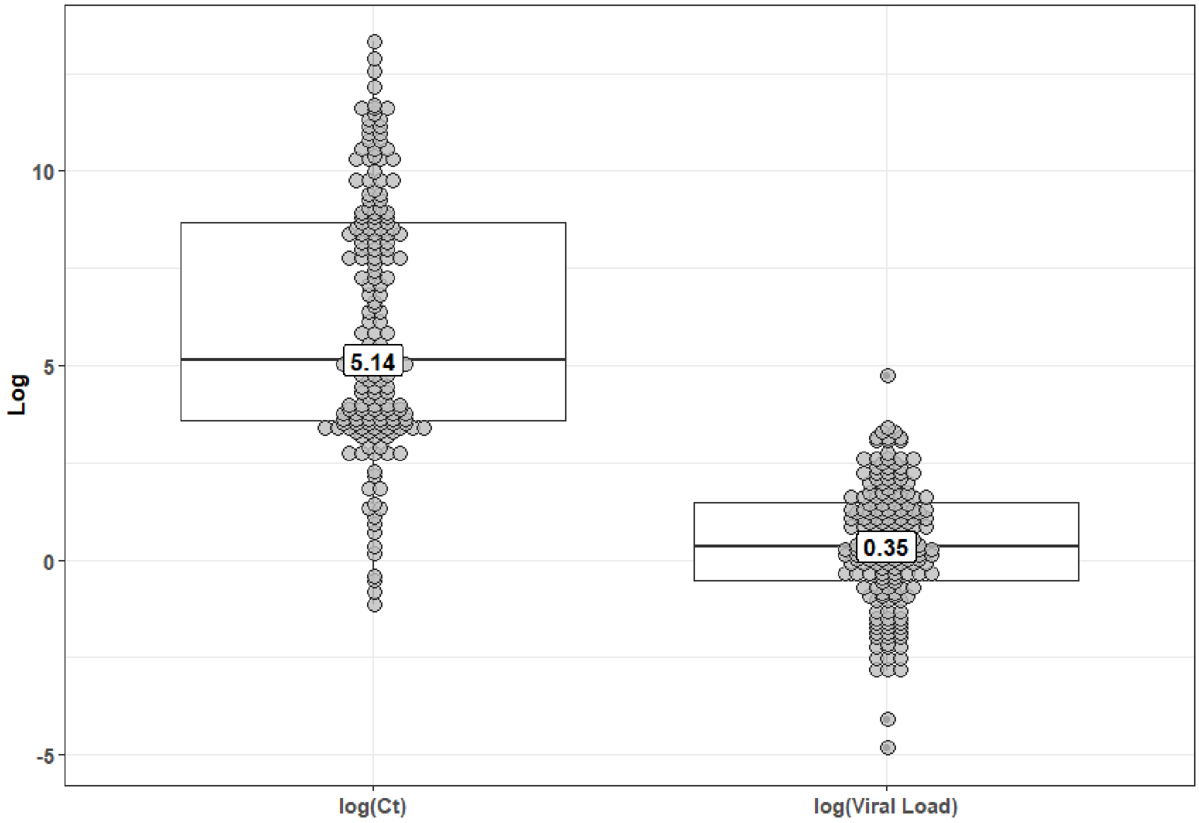

Supplementary Figure S2: Boxplot of log(Ct) and log(Viral Load). Medians are displayed in the respective boxplots.
